# Special issue: Application of biotechnology for biofuels: transforming biomass to biofuels

**DOI:** 10.1007/s13205-013-0122-8

**Published:** 2013-02-19

**Authors:** Ashutosh Mittal, Stephen R. Decker

**Affiliations:** Biosciences Center, National Renewable Energy Laboratory, Golden, CO USA

## Introduction

Rising energy prices and depleting reserves of fossil fuels continue to renew interest in the conversion of biomass to biofuels production. Biofuels derived from renewable feedstocks are environmentally friendly fuels and have the potential to meet more than a quarter of world demand for transportation fuels by 2050. Moreover, biofuels are expected to reduce reliance on imported petroleum, reduce greenhouse gas emissions, and stimulate regional economies by creating jobs and increasing demand and prices for bioproducts.

Biofuels such as ethanol are derived from food crops, biomass, or lignocellulosic materials through biochemical and thermochemical conversion processes. First-generation biofuels (i.e. corn ethanol and biodiesel) are made largely from food crops such as cereals, sugar crops, and oil seeds. The technologies to produce the first-generation biofuels from edible sugars and starches are mature and well understood, and production is primarily limited by environmental and social concerns such as competition for land and water used for food and fiber production causing increase in world commodity prices for food and animal feeds (Sims et al. 2010). Owing to these important limitations the “next-generation”, or second- and third-generation biofuels are being developed from non-edible lignocellulosic materials using advanced technologies. These lignocellulosic feedstocks include woody biomass and wood wastes, crop residues, dedicated energy crops such as switchgrass, municipal wastes, and algae. These next-generation feedstocks do not compete directly with food production and can often be produced on marginal or unused croplands. Furthermore, lignocellulosic biomass is an abundant renewable energy source, with the potential to displace a large portion of conventional energy resources such as fossil fuels and natural gas for the future production of liquid biofuels with improved environmental benefits. As a result, lignocellulosic biomass holds promise as a feedstock for a biorefinery where sugars can be transformed into building-block chemicals through fermentation, enzymatic, and chemical transformations (Ragauskas et al. 2006).

Lignocellulosic biomass is a composite structure of lignin, cellulose, and hemicellulose polymers. The efficient utilization of biomass for biofuels production requires a fractionation of biomass constituents into separate streams at maximum yields. However, a major barrier to lignocellulosic biomass utilization in any sugar platform biorefinery is its intrinsic resistance to deconstruction. This recalcitrance results from multiple factors including the heterogeneous nature of the polymer matrix, the complexity of lignin and hemicellulose spatial and chemical interactions, and the extensive hydrogen bonding of crystalline cellulose. Therefore, investigating plant cell wall biosynthesis to unravel the recalcitrant structure of lignocellulosic biomass, exploring the types of pretreatment processes used to deconstruct biomass, and developing efficient enzymatic hydrolysis are main focus areas in converting the polymeric carbohydrates present in plant biomass to fermentable sugars for cost-effective ethanol production.

## Pretreatment

In the biochemical conversion of biomass to ethanol, a pretreatment step is conducted to reduce the recalcitrance of biomass to realize the high yields vital to an economically favored commercial process. Pretreatment can be an effective method of hydrolyzing the hemicellulose or removing a fraction of the lignin, thus increasing the accessibility of the remaining cellulose to enzymatic hydrolysis and increasing enzymatic hydrolysis rates and extents. Pretreatment with mineral acids are very common, e.g. diluted sulfuric acid in a concentration range of 0.22–6 % w/w at temperatures of 100–200 °C (Esteghlalian et al. 1997; Lee et al. 1999; Hendriks and Zeeman 2009). Bases, such as sodium hydroxide (N’Diaye and Rigal 2000) and ammonia (Kim et al. 2003) are also applied to this purpose. Recently, ionic liquids have been shown to completely solubilize biomass and are considered promising solvents for the pretreatment of biomass (Shill et al. 2011). However, all of these chemicals have issues with large-scale processes. Acids are corrosive and requires the use of expensive equipment metallurgy and waste handling, bases are expensive and also require neutralization steps, ionic liquids are even more expensive at present and can be toxic at low concentrations, requiring very efficient recovery processes. All of these factors add cost and can make the overall process environmentally unfriendly and economically unattractive. Therefore, environmentally benign, low cost pretreatment technologies are constantly being investigated. As with any process involved in biomass conversion, high yields are required for maximal biomass utilization with minimized losses.

## Enzymatic hydrolysis

Due to its rigid structure and high crystallinity, cellulose provides the basic framework for plant fibers and is resistant to chemical or enzymatic hydrolysis. The enzymatic hydrolysis of cellulose is a slow process and the extent of hydrolysis is influenced by the structural properties of the biomass substrate, such as crystallinity, surface area, degree of polymerization, and porosity (Yoshida et al. 2008; Hall et al. 2010). To increase the efficiency and efficacy of the enzymatic hydrolysis process, it is generally necessary to perform a chemical pretreatment of the biomass to modify one or more of these attributes, thereby allowing better access by cellulase enzymes to cellulose. Furthermore, efficient enzymatic hydrolysis of cellulose requires the synergistic action of several cellulolytic enzymes produced by various fungal and bacterial microorganisms (Himmel et al. 2007). The mode of action of cellulolytic enzymes on cellulose chains is commonly described by the synergistic action of endo-acting enzymes that randomly cleave bonds along the cellulose chain, and processive exo-acting enzymes that degrade the polymers from chain ends (Teeri 1997; Horn et al. 2012). Despite the availability of detailed knowledge on cellulase structure and on the molecular properties and ultrastructure of cellulose, the complexity of biomass and its interactions with cellulase limit our understanding of the mechanism of efficient hydrolysis of recalcitrant cellulose. Consequently, new studies focused at understanding the mechanism for improving cellulase efficiency and productivity are at the forefront of biochemical and biotechnology research focused on transforming biomass to biofuels.

## Biomass recalcitrance

The research and development efforts of various organizations are focused on the fundamental understanding and elimination of biomass recalcitrance—the resistance of cellulosic biomass to breakdown into sugars. The development of new biomass feedstocks by altering plant cell walls via genetic manipulation of the cell wall polymer biosynthesis pathway is a very active research area. Generating cell walls with reduced recalcitrance requires detailed characterization of both plant cell wall chemistry and structure. However, to identify changes in cell wall properties resulting from genetic manipulation of specific genes in these pathways requires screening of large number of mutant plants. High throughput (HTP) analytical techniques for screening and characterizing cell wall components of large sample sizes of mutant plants are essential (Foston and Ragauskas 2012; Gjersing et al. 2012), as are rapid techniques for evaluating the susceptibility of these mutants to pretreatment and enzymatic hydrolysis (Decker et al. 2009). Recent developments have significantly advanced our ability to screen large number of plant variants for changes in recalcitrance, however these techniques require specialized equipment and expensive robotics (Selig et al. 2010). Newer, cheaper, and less complex means of measuring this recalcitrance is still needed. Similarly, understanding the cell wall composition of these same large sample sets is also a challenge. The current standard analytical technique for quantifying biomass cell wall components utilizes a two-stage acid-hydrolysis followed by high-performance liquid chromatography (HPLC) and gravimetric determinations, which are both time and labor intensive (Gjersing et al. 2012). Therefore, new HTP analytical technologies for faster and more accurate determination of cell wall components are essential to make the entire process truly high throughput and cost-effective. Consequently, the availability of simple and universal HTP analytical techniques to characterize the composition, chemical structure and morphology of a biofuel feedstock will be vital in identifying and overcoming biomass recalcitrance and producing economical biofuels and biomass-derived chemicals.

## Outlook for biotechnology research for biofuels production

Research in the production of biofuels has been growing significantly due to rising energy prices and the depletion of fossil fuels reserves. Cellulosic biomass offers an abundant and largely untapped resource for biofuels, if optimal and cost-effective conversion methodologies are determined. Biotechnology is being used to produce new advanced biofuels that perform more like gasoline, providing better fuel economy and fewer blending issues than ethanol. The scope of biofuel biotechnology spans feedstock chemistry, degradation processes, and variations in technology. To promote excellence in science and keep the global scientific and research community abreast to the recent advancements and developments taking place in the biomass conversion to biofuels area, 3 Biotech will publish a special issue of articles that focus on the “*Application of Biotechnology for Biofuels*” in 2012–2013.

This special issue addresses important recent developments related to biochemical conversion of biomass and is dedicated to advance new developments, approaches and progress in the understanding of application of biotechnology research for the efficient production of biofuels. The special issue includes original full research articles, short communications, methods and review papers.
